# Assessing sensitivity to change: choosing the appropriate change coefficient

**DOI:** 10.1186/1477-7525-3-23

**Published:** 2005-04-05

**Authors:** Paul W Stratford, Daniel L Riddle

**Affiliations:** 1 School of Rehabilitation Science, McMaster University, Hamilton ON, L8S 1C7 Canada; 2 Department of Clinical Epidemiology and Biostatistics, McMaster University, Institute for Applied Health Science, 1400 Main Street West (4th floor), Hamilton ON, L8S 1C7, Canada; 3 Department of Physical Therapy, Medical College of Virginia Campus, Virginia Commonwealth University, 1200 East Broad Street, Richmond, VA 23298-0224, USA

## Abstract

The past 20-years have seen the development and evaluation of many health status measures. Unlike the high standards demanded of those who conduct and report clinical intervention trials, the methodological rigor for studies examining the sensitivity to change of health status measures are less demanding. It is likely that the absence of a criterion standard for change in health status contributes to this shortcoming. To increase confidence in the results of these types of studies investigators have often calculated multiple change coefficients for the same patient sample.

The purpose of this report is to identify the conflict that arises when multiple change coefficients are applied to the same patient sample.

Three families of change coefficients based on different assumptions concerning the sample composition are identified: (1) the sample is homogeneous with respect to change; (2) subgroups of patients who truly change by different amounts exist; (3) individual patients, many of whom truly change by different amounts exist. We present several analyses which illustrate a major conceptual conflict: the signal (a measure's true ability to detect change) for some of these coefficients appears in the noise term (measurement error) of the others.

We speculate that this dilemma occurs as a result of insufficient preparatory work such as pilot studies to establish the likely change characteristic of the patient population of interest. Uncertainty in the choice of change coefficient could be overcome by conducting pilot studies to ascertain the likely change characteristic of the population of interest. Once the population's change characteristic is identified, the choice of change coefficient should be clear.

## Review

The past two decades have seen considerable interest in the development and evaluation of health status outcome measures [1-14]. Although the assessment of reliability and cross-sectional validity is straightforward, the same cannot be said about the evaluation of a measure's ability to detect change. Investigators have often expressed uncertainty in the choice of study design and analysis, and statements such as the following are common: "Because there is not yet agreement on the optimal design and analysis strategies for a responsiveness study, the authors evaluated the responsiveness of the FRI and RM-18 using two methods" [10]; "A variety of statistics have been used to assess responsiveness and no single one is superior" [2]; and "The purpose of this study was to determine if different indices of responsiveness provided similar rank orderings of scales in terms of responsiveness" [5]. It is likely that the absence of a gold standard for change in health status plays a prominent role in stimulating uncertainty in the choice of analysis. The solution to the expressed conundrum has often been the application of a "shotgun analysis" where multiple change coefficients are applied to a common dataset [2,4,5,9-14]. In this paper we provide a brief review of prominent study designs and change coefficients, and illustrate the conflict in applying change coefficients from different "families of analytic methods" to the same data.

### Methodological shortcomings

The methodological sophistication and standards for reporting clinical intervention trials stand in sharp contrast to those evident for longitudinal validity studies of sensitivity to change. Agencies funding clinical trials demand a clearly stated research question, evidence – often in the form of a pilot study – supporting the sample size, and a statement justifying the analysis. Journal editors require equal clarity and rigor when manuscripts pertaining to clinical trials are considered for publication. All too frequently reports of sensitivity to change of various health status measures appear to be "studies of opportunity," rather than carefully planned investigations. Notably absent from many studies are a clear statement of purpose, elaboration of design details including the expected extent to which the sample's true change is likely to be homogeneous or heterogeneous (we will subsequent refer to this as the sample's change characteristic), justification of sample size, and a commitment to the most appropriate analysis [2,5,6,9,11,14,15]. The importance of specifying the change characteristic of the sample is that it dictates the choice of change coefficient, or at least the family from which the change coefficient will be selected.

### Study designs and sample change characteristics

Previous monographs have provided comprehensive reviews of popular designs for sensitivity to change studies [16-18] and it is not our intent to repeat these discussions. However, to set the stage we identify three popular designs and their corresponding samples' change characteristics: (1) patients who are expected to truly change by approximately the same amount are assessed at two points in time [1]; (2) two or more identifiable subgroups of patients who are expected to change by different amounts are assessed at two points in time [19]; and (3) patients, many of whom, are expected to truly change by different amounts are assessed at two points in time [20]. To distinguish between Designs 1 and 3 we will refer to Design 1 as being homogeneous and Design 3 as being heterogeneous with respect to change. Design 2 shares the characteristics of Designs 1 and 3. Consistent with Design 1 is the assumption that patients within a subgroup truly change by approximately the same amount and the extent to which differences occur is attributed to measurement error. Like Design 3, the ability of a measure to detect true change is reflected by the extent to which the measure is capable of differentiating the amount of change between units that truly change by different amounts. The units are groups of patients for Design 2 and individual patients for Design 3.

Although the three study designs are conceptually simple, ascertaining a sample's change characteristic is more demanding. Perhaps the most popular method, particularly for Designs 2 and 3, has been the retrospective global rating of change [4,5,13,14,21]. Here, at the follow-up assessment patients provide their impression of global change in addition to completing the measure of interest. This single item global rating of change is then used as the standard for assessing the measure's ability to detect change. Norman and colleagues [22] have challenged this approach on three counts: (1) the notion that the measurement properties of the single item global rating are superior to the multi-item measure under investigation; (2) judgments of change are psychologically difficult and therefore suspect; and (3) correlated measurement error between the global rating and the measure under investigation inflates the true association between the two ratings. With respect to the last point, Norman et al [22], showed that the retrospective global rating of change can result in declaring a measure responsive in a sample of stable patients.

An alternative to the retrospective rating is the prognostic rating of change [19,23-25]. This approach is not subject to errors of recall or correlated error; however, it is dependent on the ability of the rater to accurately estimate the extent of change that might occur. As the name suggests, the essential feature of the prognostic rating method is an a priori declaration of the sample's change characteristic. Sensitivity to change studies have applied three designs using prognostic ratings of change: (1) randomized trials where interventions of known effectiveness are compared to placebo or weaker interventions [19]; (2) cohort studies where a known prognostic variable is used to classify patients into groups that are expected to change by different amounts [25]; and (3) clinicians assign expected change scores to patients at their initial visit [23,24]. Meenan et al [19], in a three group (placebo, oral gold, injectable gold) randomized controlled trial, investigated the sensitivity to change of the Arthritis Impact Measurement Scales and several other clinical measures. Consistent with a priori hypotheses, the measures demonstrated a gradient in treatment effects with the injectable gold group demonstrating the greatest change and the placebo group showing least change. Stratford and Binkley [25] applied a cohort design where the natural history of patients with low-back pain was used to established two groups of patients with different change characteristics. Specifically, these investigators theorized that patients with low-back pain of less than 2-weeks duration would change more over the subsequent 2-week interval than would patients who presented with low-back pain of 2 or more weeks duration. Westaway et al [23] investigated the sensitivity to change of the Neck Disability Index (NDI) [26] and Patient Specific Functional Scale (PSFS) [27]. These investigators theorized that seasoned clinicians' would be able to distinguish among patients who would change by different amounts over an interval of several weeks. At the initial assessment clinicians rated patients' prognoses on a 5-point scale. Prognostic ratings were based on clinical judgment alone. The results demonstrated significant correlations between the prognostic rating of change and the measures' change scores.

### Study designs and their respective families of analytic methods

Sensitivity to change studies are rich with descriptions of change coefficients [2,5,6,9-12,15] which we place in the following three groups or families according to study design: Design 1, coefficients based on homogeneity of patients change characteristics; (2) Design 2, between group contrast coefficients; (3) Design 3, correlation coefficients.

### Homogeneous patient change

This design and analysis is based on the premise that the sample consists of patients who are expected to change by approximately the same amount over the study period. Of interest is not what accounts for the change – it could be the natural history or the application of an effective intervention – but rather that the amount of change is expected to be reasonably homogeneous among patients. The ability of a measure to assess change is quantified by dividing the mean change (signal) by the variation in change or sample characteristics at baseline (noise). The standardized response mean (SRM = mean change/standard deviation of change) [1] is a frequently reported change coefficient associated with this design. Statistical tests include the paired t-test and repeated measures ANOVA with one within patient factor (occasion at 2 levels: baseline and follow-up) and no between patient factor. Of the three designs, this one is considered to be the weakest because it does not challenge a measure's ability to discriminate among different amounts of change [16,17].

### Heterogeneous patient composition: between group contrast

This design is based on the premise that identifiable subgroups of patients who change by different amounts exist. Change coefficients include area under receiver operating characteristic (ROC) curves [18] and Norman's S_repeat _[28]. Statistical analyses for this design include the z-statistic for the area under a ROC curve [16], t-test for independent sample means of change scores, and repeated measures analysis of variance (ANOVA) with one within patient factor (occasion at 2 levels) and one grouping factor (amount of change at 2 or more levels: small change, large change) [28].

### Heterogeneous patient composition: among patient contrast

Like the first design, this one investigates a single group of patients. However, rather than the patients being reasonably homogeneous with respect to change, the patients are expected to truly change by different amounts. Moreover, an essential aspect of this design is that an external standard is applied, the change scores of which are compared to the change scores of the measure of interest. A measure's ability to detect change is based on a correlation analysis [5,16,17].

### Problem clarification

Investigators have often applied analyses and change coefficients from the three families of tests to the same patient sample [4,6,9,11], apparently without realizing that the coefficients are based on different, and at times conflicting assumptions concerning the sample's change characteristic. For example, Kopec et al [4] reported a study that was conceived to "determine whether the Quebec scale (a functional status measure for patients with low-back pain) is a reliable, valid, and responsive measure of disability, in back pain, and to compare it with other disability scales." The sample was diverse in that it included patients from physical therapy clinics, physiatry centers, rheumatology clinics, family practice groups, and pain clinics. Statistical tests included the paired t-test, repeated measures ANOVA with one grouping factor (amount of change), and a correlation of the Quebec's change scores with those of a retrospective global rating of change. Change coefficients included the SRM [1], Norman's S_repeat _[28], and an unnamed correlation coefficient. The three analyses were applied to the same group of patients. To underscore the theoretical conflict in applying these coefficients to the same patient sample we will link the coefficients reported by Kopec et al [4] through repeated measures and regression ANOVA tables.

### Illustrative comparison of change coefficients

To facilitate discussion, we will make reference to the dataset displayed in Table 1. These data represent the results from a hypothetical study where a health status measure was administered to 20 patients at their baseline assessment and at follow-up 2-months later. The investigator believed that patients would improve over this interval. Also, at the follow-up visit patients provided a global rating of change on a 15-point scale (-7 to 7) [21]. Furthermore, the investigator dichotomized the patients' global ratings using a cut-point of 5 on the global rating. The investigator did not declare detailed a priori assumptions concerning the extent to which patients were expected to change by different amounts. Three analyses are presented: (1) a repeated measures ANOVA with no grouping factor and 1-within patient factor; (2) a repeated measures ANOVA with 1-grouping factor and 1-within patient factor; and (3) a correlation of the measure's change scores with those of the retrospective global rating of change. Although our illustration represents a hypothetical study, the design and analyses are consistent with the approach of Kopec et al [4] and many other studies reported in the literature [6,9,12].

**Table 1:** Summary of synthetic data

**Patient ID**	**Initial Assessment**	**Follow-up Assessment**	**Change Initial-follow-up**	**Global Rating**	**Global Rating Dichotomized**
1	16	14	2	3	0
2	14	14	0	1	0
3	13	5	8	6	1
4	19	10	9	5	1
5	14	8	6	6	1
6	3	4	-1	2	0
7	18	12	6	3	0
8	8	6	2	5	1
9	10	5	5	7	1
10	12	9	3	6	1
11	14	10	4	5	1
12	9	9	0	3	0
13	10	8	2	6	1
14	14	10	4	4	0
15	17	16	1	1	0
16	9	6	3	2	0
17	9	8	1	1	0
18	13	7	6	6	1
19	8	4	4	4	0
20	13	8	5	7	1
					
Mean	12.15	8.65	3.50	4.15	
St. dev.	3.92	3.38	2.71	2.03	

### Homogeneous patient change analysis

The first analysis presented is a repeated measures ANOVA with no grouping factor and 1-within patient factor, occasion, at 2-levels (baseline and follow-up) [29]. The results from this analysis are shown in Table 2. The statistical analysis is equivalent to a paired t-test and the F-value of 33.49 is equal to the square of the paired t-value. The SRM [1] is typically defined as:

**Table 2:** Repeated measures ANOVA with one within patient factor and no grouping factor

**Source**	**DF**	**SS**	**MS**	**F, (p)**
Between Patients	19	439.60	23.14	
**Within Patients**	**20**	**192.00**		
Occasion	1	122.50	122.50	33.49, (<0.001)
Error	19	69.50	3.66	



However, it can also be calculated from the repeated measures ANOVA shown in Table 2:



where MSO is the mean square occasions, MSE is the mean square error, and n is the number of patients.

### Heterogeneous patient composition: between group analysis

This analysis is based on a repeated measures ANOVA with 1-between patient grouping factor at 2-levels (amount of change: a small amount or a large amount according to the dichotomized retrospective global rating of change) and the same within patient grouping factor as in the previous analysis [29]. The results are reported in Table 3. The group-by-occasion interaction term represents the extent to which the two groups changed by different amounts. The F-value for this term, F_1,18 _= 8.62, is the square of the t-value that would have been obtained had a t-test for independent sample means based on change scores been applied. Norman's S_repeat _[28] is calculated from the following information provided in Table 3:

**Table 3:** Repeated measures ANOVA with one within patient factor and one grouping factor

**Source**	**DF**	**SS**	**MS**	**F, (p)**	**Variance**
**Between Patients**	**19**	**439.60**			
Group	1	3.60	3.60		
Error	18	436.00	24.22		
**Within Patients**	**20**	**192.00**			
Occasion	1	122.50	122.50	46.92, (<0.001)	
Group by occasion	1	22.50	22.50	8.62, (0.009)	1.99
Error	18	47.00	2.61		2.61



### Heterogeneous patient composition: among patient analysis

This analysis represents a correlation of change scores with patients' retrospective global ratings of change. To show the location of the sources of variation, we generated the correlation coefficient from a regression analysis [30]. Also, we provide an intermediate analysis which replicates the previous identifiable subgroup analysis. Here, "group" was coded as a dummy variable (0 or 1): it is the dichotomized rating of change shown in Table 1. Notice that the F-value in Table 4 is identical to that for the group-by-occasion interaction term reported in Table 3. Table 5 presents the results from the correlation of change scores with the raw retrospective global ratings of change.

**Table 4:** Regression analysis with group as a dummy variable

**Source**	**DF**	**SS**	**MS**	**F, (p)**	**Correlation**
Regression	1	45.00	45.00	8.62, (0.009)	r = 0.57
Residual	18	122.80	6.82		

**Table 5:** Regression analysis with raw global rating change scores

**Source**	**DF**	**SS**	**MS**	**F, (p)**	**Correlation**
Regression	1	56.30	56.30	12.25, (0.003)	r = 0.64
Residual	18	139.00	4.60		

### Source of conflict among analyses

An examination of the sum of squares terms (SS) in the ANOVA tables exposes the deficiency in applying these tests to the same dataset. Notice that when a repeated measures ANOVA with no grouping factor is applied, its SS error term contains both the group-by-occasion interaction term and the residual error from the repeated measures ANOVA with a grouping factor. Thus, to the extent that identifiable subgroups of patients exist, their presence drives down the magnitude of the SRM: the signal has become noise. The regression analyses reveal that this phenomenon extends to situations where patients truly differ in their change scores. Moreover, a comparison of the two regression analyses and correlation coefficients demonstrates that to the extent individual differences in change scores truly exist among patients, a between group analysis will under-estimate the ability of a measure to detect change.

### Reasons for "Agreement" among coefficients

A natural question is if the signal for the between group and among patient change scores is contained in the noise portion of the SRM, how is it possible to obtain a change coefficient that differs from zero for this analysis? There are at least three answers.

First, the reported coefficients may not truly differ from zero. This statement is based on the observation that change coefficients are often presented as point estimates [2,4,5]. Without knowledge of a confidence interval or hypothesis test, one cannot ascertain the chance that a reported point estimate truly differs from zero. As a matter of interest, the 95% confidence interval for the reported SRM of 1.29 in our example is 0.91 to 1.92, confirming that it is highly likely that it differs from zero.

The second explanation considers a situation similar to that of our data where the SRM is greater than zero. Although self-evident, it is important to acknowledge that investigators interested in evaluating a measure's ability to detect change select patients who, in most instances, are expected to truly improve. Accordingly, the mean change for the group will be greater than zero even when some patients remain stable or get worse. When the mean change is greater than zero, the SRM will be greater than zero, even when subgroups or individual patients truly change by different amounts.

The third explanation addresses the situation where apparent patient differences in change scores, as represented by a correlation with another measure, are observed in a sample that is truly homogeneous with respect to change. In this case the design premise applied most frequently by investigators is that change scores on the measure under investigation will correlate with patients' retrospective global ratings of change. To the extent that in clinical practice clinicians ask patients about their perceptions of change, this methodology seems reasonable. However, the major limitation associated with this approach is that it spuriously inflates the observed correlation coefficient. To understand the mechanism of this apparent association, a brief review of the relationship and assumptions of observed, true, and error scores is necessary [31]. In this example, the observed scores are those reported by patients on the measure under investigation and the retrospective global rating of change. True scores are unknown values that represent the scores that would be obtained in the absence of measurement error. Error scores are the differences between observed scores and true scores. The framework for comparing the change scores of a measure to the global rating of change is that of parallel assessments of the same attribute. A fundamental assumption is that the measure's error scores and the global rating's error scores are uncorrelated [31]. However, it is extremely unlikely that the error scores are independent when a patient provides both the measure's change score and that of the global rating [22]. The consequence is that the observed correlation will be greater than zero even when the correlation between the true scores is zero.

## Conclusion

The absence of a gold standard combined with multiple change coefficients has created uncertainty for those who investigate the sensitivity to change of health status measures. In an attempt to increase confidence in a measure's ability to detect change investigators have often reported multiple change coefficients derived from the same patient sample, the belief being that uniform findings among coefficients adds to the confidence in the results. We contend that this approach is inconsistent with theory: the signal for some coefficients is included in the noise of others. We suggest that rather than calculating multiple change coefficients, a more theoretically sound approach is to devote more preparatory work to determine the likely change characteristics of the patients of interest. Once the sample's change characteristic is established, the choice of change coefficient should be clear. Moreover, when the opportunity presents, investigators are encouraged to select the more rigorous designs which not only allow the assessment of change, but also challenge a measure's ability to differentiate among patients or groups of patients who change by different amounts.

## Conflict of interest

The author(s) declare that they have no competing interests.

## Authors' contributions

Both authors contributed to the conceptualization and writing of this manuscript.
